# A novel model of ischemia in rats with middle cerebral artery occlusion using a microcatheter and zirconia ball under fluoroscopy

**DOI:** 10.1038/s41598-021-92321-w

**Published:** 2021-06-17

**Authors:** Teppei Komatsu, Hiroki Ohta, Haruhiko Motegi, Junichi Hata, Koshiro Terawaki, Makoto Koizumi, Kanako Muta, Hirotaka James Okano, Yasuyuki Iguchi

**Affiliations:** 1 Department of Neurology, The Jikei University School of Medicine, 3-25-8 Nishishimbashi, Minato-ku, Tokyo, 105-8461 Japan; 2 Division of Regenerative Medicine, Research Center for Medical Sciences, The Jikei University School of Medicine, Tokyo, Japan; 3 Department of Radiological Science, Graduate School of Human Health Sciences, Tokyo Metropolitan University, Tokyo, Japan; 4 Laboratory Animal Facilities, Research Center for Medical Sciences, The Jikei University School of Medicine, Tokyo, Japan; 5 Laboratory of Veterinary Surgery, Graduate School of Agricultural and Life Sciences, The University of Tokyo, Tokyo, Japan

**Keywords:** Stroke, Brain, Imaging techniques

## Abstract

The failure of neuroprotective treatment-related clinical trials may be partially caused by unestablished animal models. Existing animal models are less likely to provide occlusion confined to the middle cerebral artery (MCA), making transarterial intervention difficult. We aimed to develop a novel focal stroke model using a microcatheter and zirconium dioxide that is non-magnetic under fluoroscopic guidance, which can monitor MCA occlusion and can improve hemorrhagic complications. Using male Sprague Dawley rats (n = 10), a microcatheter was navigated from the caudal ventral artery to the left internal carotid artery using an X-ray fluoroscopy to establish local occlusion. All rat cerebral angiographies were successful. No rats had hemorrhagic complications. Eight (80%) rats underwent occlusion of the MCA bifurcation by zirconium dioxide. Accidentally, the left posterior cerebral artery was failure embolized in 2 rats (20%). The median operating time was 8 min. All rats of occlusion MCA revealed an incomplete hemiparesis on the right side with neurological deficit score ranging from 1 to 3 (median 1, interquartile range 1–3) at 24 h after the induction of ischemia. Moreover, 2% 2,3,5-triphenyl tetrazolium chloride staining showed that the median infarct volume (mm^3^) was 280 (interquartile range 267–333) 24 h after the left MCA bifurcation occlusion. We present a novel rat model for focal stroke using a microcatheter and zirconium dioxide which does not affect the MRI. The model is predictable which is well confined within the territory supplied by the MCA, and reproducibility of this model is 80%. Fluoroscopy was able to identify which the MCA occlusion and model success while creating the model. It permitted exclusion of animals with complications from the experiment.

## Introduction

Stroke is the second most common cause of death and the third most common cause of disability-adjusted life years lost worldwide^1^. In most patients, ischemic stroke results from occlusion of the middle cerebral artery (MCA). As such, rodent models have been developed to mimic human focal ischemic stroke^2^. The most widely used rat model for focal cerebral ischemic stroke involves occlusion of the MCA (MCAO) by the intraluminal suture method, in which a nylon thread is blindly inserted from the external carotid artery (ECA)^3,4^. The main limitation of this model is that blood flow to the posterior cerebral artery (PCA) and branches of the internal carotid artery (ICA) are obstructed, resulting in large and variable infarctions of the cortical and subcortical areas^5–11^. Besides, the procedure is very invasive because the ECA is ligated after the neck incision. Ligation of the ECA results in ischemic necrosis of the mastication and hypopharyngeal muscles, which negatively affects behavioral testing outcomes^12,13^. There are additional models for inducing stroke, all with merits and limitations (Table 1)^3,4,14–19^.

**Table 1:** Comparison between the most prevalent rodent stroke models and the model in the current study.

	Suture model^3,4^	Thromboembolic model^19^	Macrosphere model^14,15^	Current model
Procedure	Incision	Incision	Incision	Puncture
Approach site	Neck	Neck	Neck	Tail
Affected ischemic area	MCA/ECA/AChA/VTA/HTA/PCA	MCA/ECA	MCA/ECA/AChA	MCA
Identify the vessel occlusion while creating the model	Impossible	Impossible	Impossible	Possible
Identify model success while creating the model	Impossible	Impossible	Impossible	Possible
Model failure rate	2/12 (17%)^14^	6/26 (23%)^19^	3/13 (23%)^14^	2/10 (20%)
24-h mortality	1/10 (10%)^14^	1/26 (4%)^19^	1/10 (10%)^14^	0/10 (0%)
SAH rate	8–30%^14,16–18^	4/26 (15%)^19^	0/13 (0%)^14^	0/10 (0%)
Reperfusion stroke model	Possible	Possible	Impossible	Impossible

*AChA* anterior choroidal artery, *ECA* external carotid artery, *HTA* hypothalamic artery, *MCA* middle cerebral artery, *PCA* posterior cerebral artery, *SAH* subarachnoid hemorrhage, *VTA* ventral thalamic artery.

There has been growing interest regarding the use of regenerative medicine for sequelae of cerebral infarctions, and clinical trials on intravenous administration of bone marrow mesenchymal cells have commenced. However, intravenous administration may result in the medicine being taken into organs other than the target, such as the lungs, thereby reducing the therapeutic effect. Therefore, it is necessary to urgently develop a highly reproducible animal model that can verify intra-arterial cell administration for cerebral infarctions.

Here, we present a highly selective rat MCAO model by percutaneous caudal arterial puncture using a microcatheter and zirconia ball under fluoroscopic guidance. The model is predictable and reproducible, which is well confined within the territory supplied by the MCA, and the surgical procedures can be typically completed within 10 min. Furthermore, minimally invasive and highly reproducible procedure lead to a “reduction” in the suffering and “refinement” of the welfare of laboratory animals.

## Materials and methods

The authors declare that all supporting data are available within the article.

### Animal model and protocol

Male normotensive adult Sprague Dawley rats (n = 10; 11–27 weeks of age; 366–587 g on the day of surgery) obtained from Nihon SLC (Japan SLC, Inc. Shizuoka, Japan) were housed in polycarbonate cages under temperature-controlled conditions (temperature: 24–25 °C; relative humidity: 50–60%) with a 12-h light–dark cycle. All rats had free access to water and pelleted food (CE-2, CLEA Japan, Inc., Gifu, Tokyo, Japan). Our study was approved by the Institutional Animal Care and Use Committee of the Jikei University School of Medicine (protocol number: 2016-105). All procedures were conducted according to the Fundamental Guidelines for Proper Conduct of Animal Experiments and Related Activities in Academic Research Institutions issued by the Japanese Ministry of Education, Culture, Sports, Science, and Technology. This study was carried out in compliance with the ARRIVE guidelines. This study doesn’t need the control group because of making stroke model study. Anesthesia was maintained with 1–3% isoflurane through a vaporizer for small experimental animals and a facial mask. Body temperature was maintained during surgery at 36–37.5 °C using a multi-panel heater (Vivaria, Osaka, Japan). The animal’s body, including the entire tail, must be kept warm, as previously reported^20,21^. No preparation, such as shaving, disinfection, or antibiotics, is required.

**Figure 1:**
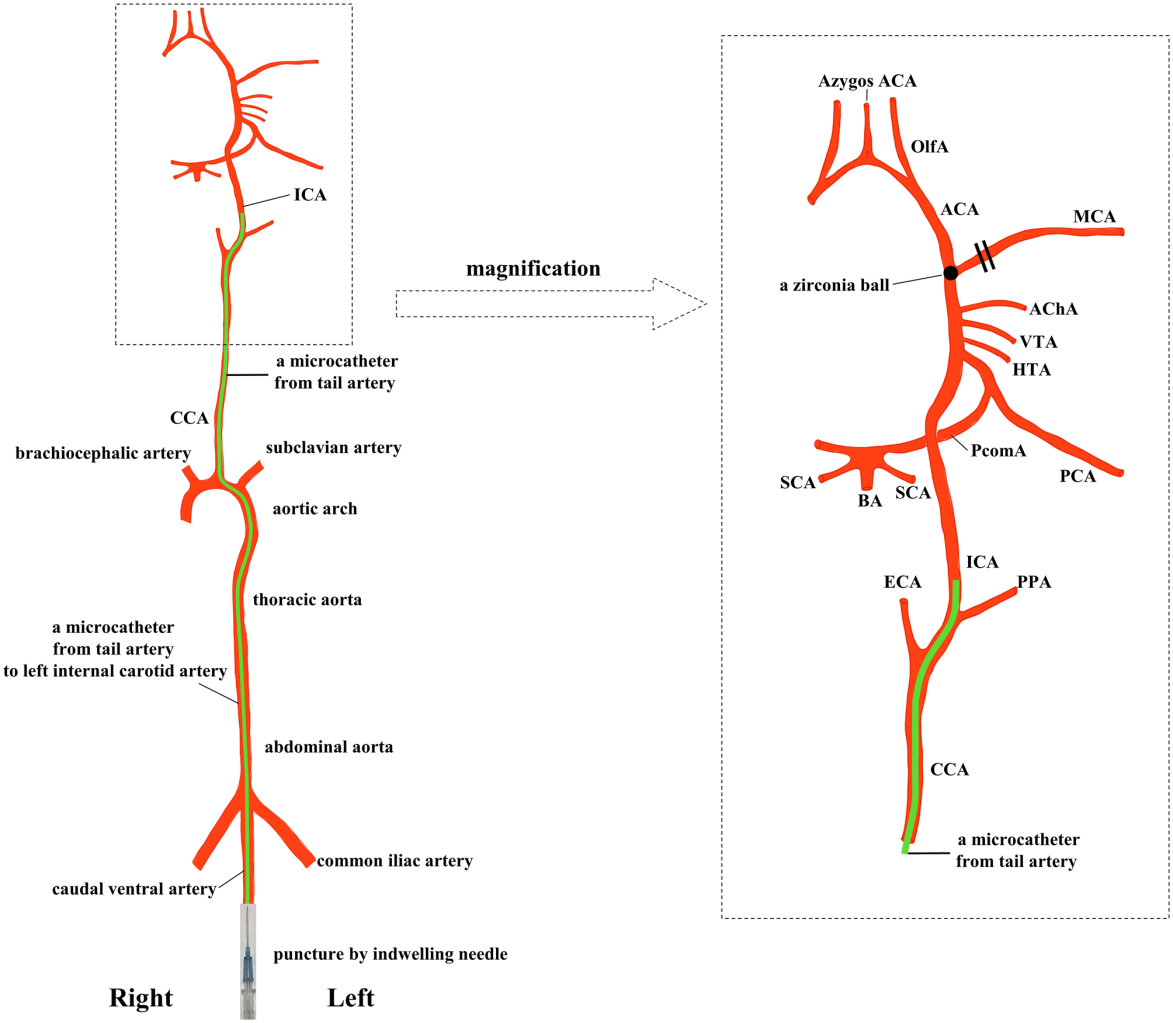
Schematic representation of the major rat arteries (view from the ventral) and the appropriate zirconia ball position during occlusion of the MCA. The microcatheter is inserted into ICA through caudal artery, aorta and CCA using fluoroscopic imaging. One zirconia ball is advanced in the microcatheter by slow injection and selectively occlude the ACA-MCA bifurcation. The zirconia ball block bold flow to the MCA, but not to the AChA, VTA and HTA. *ACA* anterior cerebral artery, *AChA* anterior choroidal artery, *BA* basilar artery, *CCA* common carotid artery, *ComACA* common (azygos) anterior cerebral artery, *ECA* external carotid artery, *HTA* hypothalamic artery, *ICA* internal carotid artery, *MCA* middle cerebral artery, *OlfA* olfactory artery, *PCA* posterior cerebral artery, *PcomA* posterior communicating artery, *PPA* pterygopalatine artery, *SCA* superior cerebellar artery, *VTA* ventral thalamic artery.

**Figure 2:**
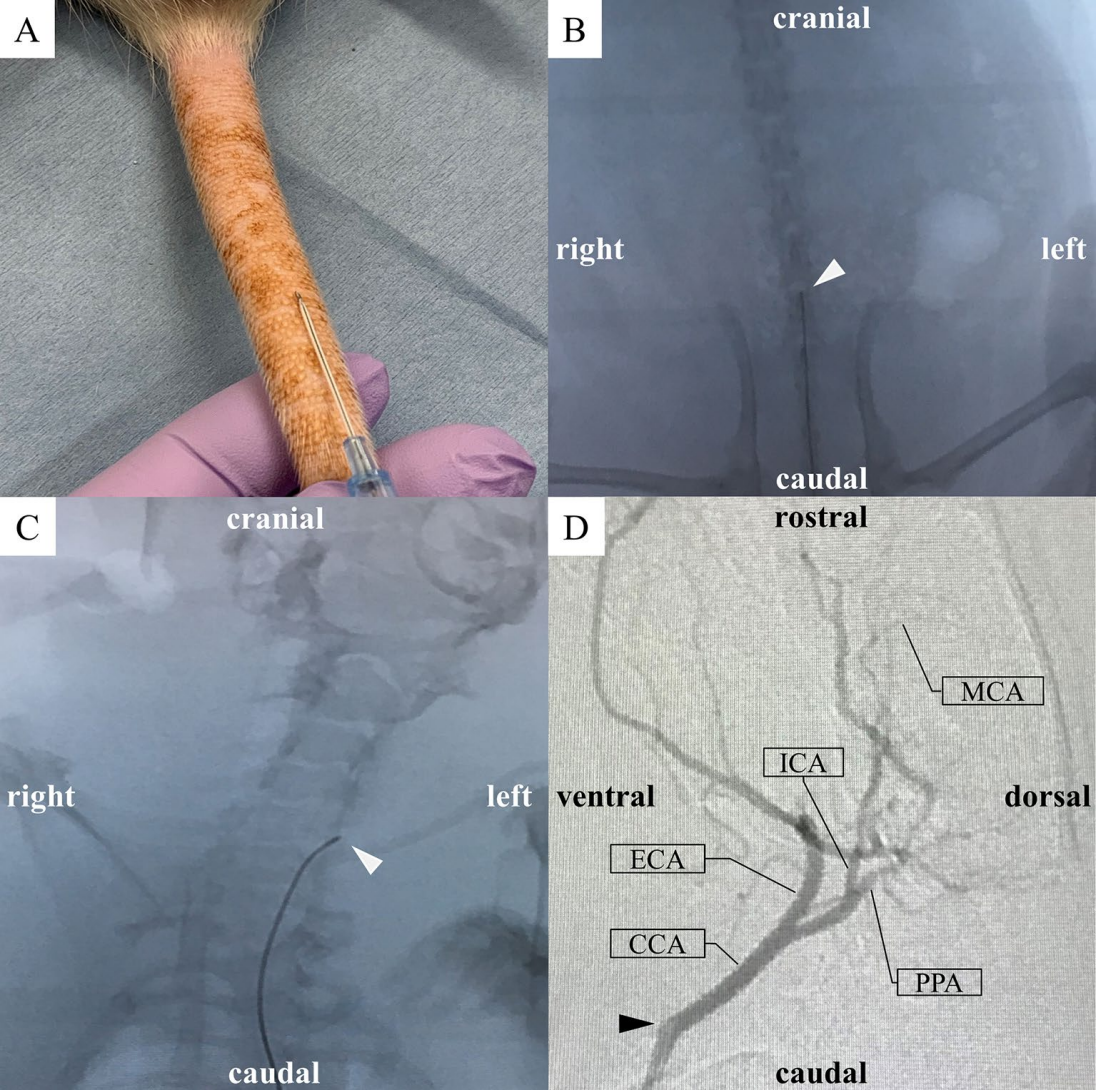
The minimally invasive intra-arterial approach under fluoroscopic guidance. (**A**) Puncture of the caudal ventral artery in a rat. An indwelling needle was inserted through the ventral midline, approximately 5 cm from the root of the tail, at a sharp angle. (**B,C**) Rat fluoroscopic images of the microcatheter and wire (arrowhead) inserted carefully through the plastic outer indwelling needle, and then inserted into the caudal ventral artery and guided into the abdominal aorta (**B**) and the left CCA (**C**). (**C**) The C-arm was tilted at a 20° LAO angle for easy cannulation of the left CCA. (**D**) Left CCA angiography (LAO angle = 80°). The arrowhead shows the tip of the microcatheter. *CCA*; common carotid artery, *ECA* external carotid artery, *ICA* internal carotid artery, *LAO* left anterior oblique, *MCA* middle cerebral artery, *PPA* pterygopalatine artery.

**Figure 3:**
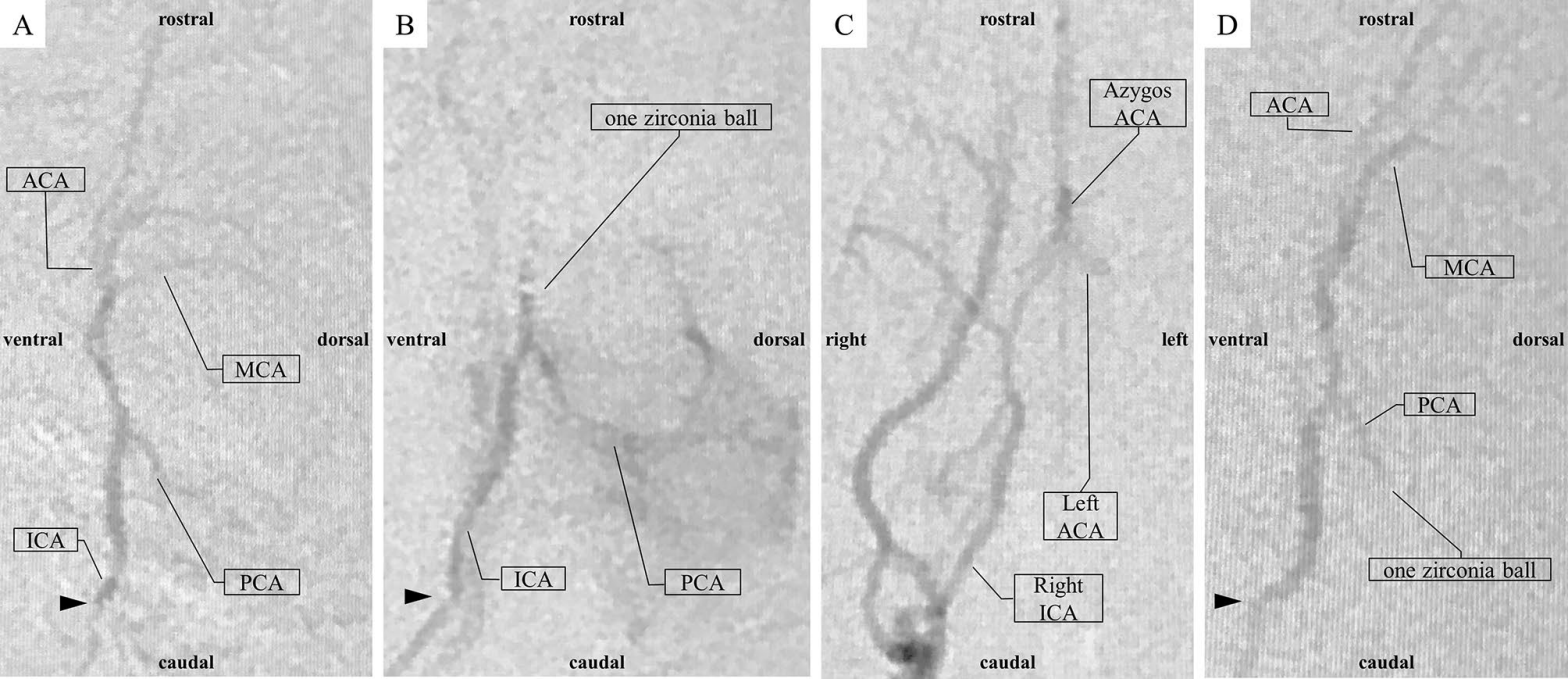
Angiography and embolization. The arrowhead shows the tip of the microcatheter. (**A**) Left ICA angiography (LAO angle = 80°). (**B**) The left ACA and MCA bifurcation was selectively embolized by one zirconia ball (LAO angle = 80°). The PCA was better visualized than before embolization. (**C**) Right CCA angiography (LAO angle = 0°). The left ACA retrograde flow is maintained through the right ACA. (**D**) The left PCA was accidentally failure embolized by one zirconia ball (LAO angle = 80°). *ACA* anterior cerebral artery, *ICA* internal carotid artery, *LAO* left anterior oblique, *MCA* middle cerebral artery, *PCA* posterior cerebral artery.

**Figure 4:**
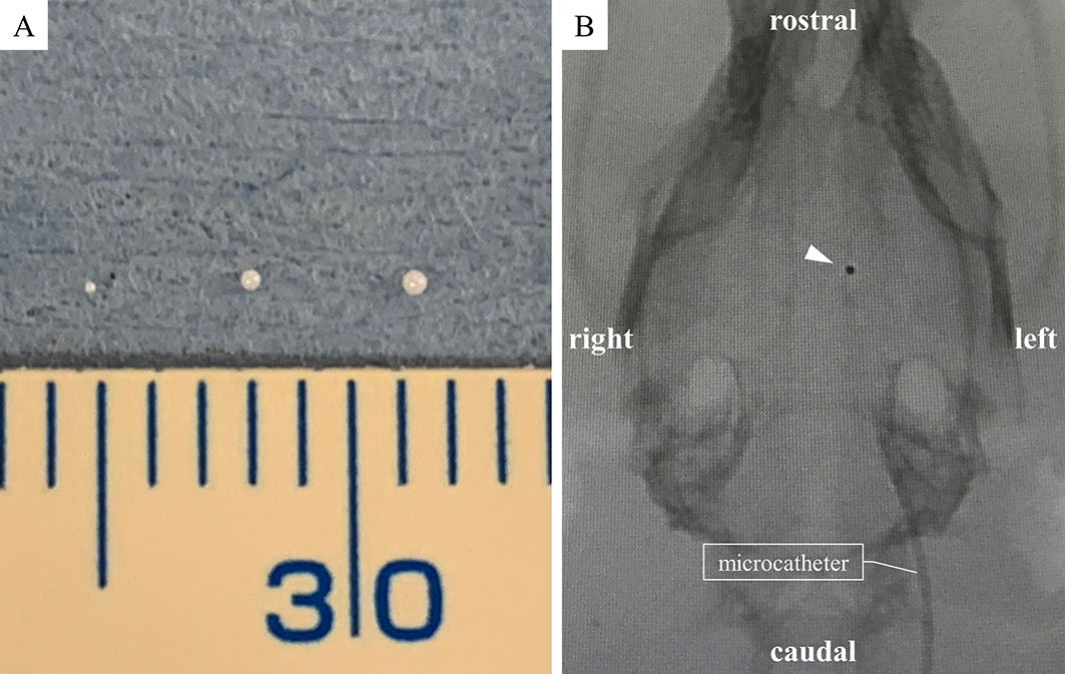
(**A**) Three sizes of zirconia ball. From left to right: 0.2 mm, 0.4 mm and 0.5 mm in diameter. The zirconia ball used in the stroke model was 0.4 mm in diameter. (**B**) Fluoroscopy shows the anterior cerebral artery-middle cerebral artery bifurcation that was selectively embolized by one zirconia ball 0.4 mm in diameter (arrowhead). The site of embolization can be easily identified during fluoroscopy because of the high visibility of zirconia ball.

Major arteries of rat with microcatheter and zirconia ball and a simplified operation scheme are shown in Fig. 1^22–24^. The polyamide microcatheter (inner diameter (ID) 0.42 mm, outer diameter (OD) 0.55 mm, Kaneko Cord, Tokyo, Japan) was first flushed with heparinized physiological saline. The rat was placed in a supine position with a stretched tail. Indwelling needle (venous indwelling needle for human, 22 gauge, SR-OT2225C, TERUMO, Tokyo) was inserted through the ventral midline artery approximately 5 cm from the root of the tail of the rat (Fig. 2A). After confirmation of arterial blood backflow from the indwelling needle, a microcatheter and wire (OD 0.4 mm, FGW16-AG18S30, Toray Medical, Tokyo, Japan) were inserted as long as no resistance was felt. Upon resistance, the insertion of the microcatheter was stopped immediately, and the position of the microcatheter was confirmed using fluoroscopic imaging. The wire and microcatheter were guided from the caudal ventral artery to the abdominal aorta (Fig. 2B), the aortic arch, and the left common carotid artery (CCA) (Fig. 2C) using an X-ray fluoroscopic imaging (Artis Zee, Siemens, Germany). Then, the wire was carefully removed so as not to change the position of the microcatheter. The contrast media (Iohexol, Daiichi Sankyo, Tokyo, Japan) at half concentration was rapidly injected from the microcatheter. Then, left CCA angiography was performed, in which 0.1 ml of the contrast media was injected using an X-ray fluoroscopy (Fig. 2D). The bifurcation of the ICA branch was visualized by tilting the C-arm at an LAO angle of 80° (Fig. 2D). The contrast images were overlaid for improving visualization, and the microcatheter was guided to the branch of the left ICA. Left ICA selective angiography could then detect the left anterior cerebral artery (ACA), MCA, and PCA (Fig. 3A). Then, one zirconia ball (zirconium dioxide, 0.4 mm in diameter; Nikkato, Tokyo, Japan). Figure 4A was advanced in the microcatheter by slow injection of approximately 0.1 ml of heparinized physiological saline (Supplemental File—Videos 1, 2), and the ACA-MCA bifurcation was selectively embolized (Figs. 3B, 4B). The zirconium dioxide does not affect the MRI because of non-magnetic material. The high visibility of zirconia ball during fluoroscopy was found to be comparable to that of wire. The microcatheter was inserted into the right CCA and contrasted to confirm that the left ACA had retrograde blood flow from the right ACA and only obstructed the left MCA in the frontal view (LAO angle 0°) (Fig. 3C). The time from the puncture to model creation was measured. The plastic outer indwelling needle was fixed with sealing film (Parafilm, Bemis, United States of America) and gaffer tape and was not removed until 24 h after the embolization. Then, cerebral angiography was performed to confirm whether spontaneous recanalization had occurred. No postoperative pain relief was required.

### Neurological deficit score

Neurological function was evaluated 3, 6, and 24 h after the induction of ischemia using a 0–5-point scale neurological score, as described previously^25,26^: 0 = no neurological deficit; 1 = failure to extend right forepaw fully when lifted by tail; 2 = circling to the right; 3 = falling to the right; 4 = no spontaneous walk or in a comatose state; 5 = death. Friedman’s test was used to assess differences neurological function between 3, 6 and 24 h after the induction of ischemia.

### Cerebral angiography 24 h after the creation of the stroke model

Cerebral angiography was performed 24 h after the creation of the stroke model to determine whether spontaneous recanalization had occurred. Cerebral angiography could be performed in the same way as when the model was created without re-puncture by using the plastic outer indwelling needle that was left behind.

### Magnetic resonance imaging (MRI) analysis

MRI was performed using a 9.4-T BioSpec 94/20 (Biospin GmbH, Ettlingen, Germany) device and a transmitting coil with a 72-mm inner diameter and a rat brain size 4 channel receiver coil immediately before the rats were euthanized. T2-weighted imaging and MR angiography were performed using the time of flight theory. T2-weighted imaging was performed using the rapid acquisition with a relaxation enhancement pulse sequence^27^. The following parameters were set: repetition time: 5000 ms, effective echo time: 42.0 ms, flip angle: 90°, RARE factor: 8, field of view: 20 × 20 mm^2^, matrix size: 200 × 200, image resolution: 100 × 100 μm, and slice thickness: 800 μm. The total T2-weighted imaging scan time was 6 min and 15 s. MR angiography was performed using a three-dimensional fast low angle shot pulse sequence^28^. The following parameters were set to achieve the time of flight effect: repetition time: 14 ms, echo time: 2.28 ms, flip angle: 8°, number of average: 6, field of view: 20 × 18 × 22 mm^2^, matrix size: 166 × 148 × 180, and image resolution: 122 × 122 × 122 μm. The total scan time of the three-dimensional fast low angle shot pulse sequence was 36 min 20 s. MR angiography was reconstructed using the maximum intensity projection method in ParaVision version 6.0.1 software (Bruker, Ettlingen, Germany). To acquire MRI data, the rats were scanned in the prone position on an imaging stretcher and administered a mixture of air and 1.5–3.0% concentrated isoflurane (Abbott Laboratories, Abbott Park, IL, USA). Respiration was regularly monitored during the scanning to manage the animal’s physical condition.

### Postmortem analysis

Twenty-four hours after embolization, the animals were deeply anesthetized and euthanized. The brains were removed and were sectioned coronally into 6 slices (thickness, 2 mm) from the olfactory bulb to the cerebellum and then stained with 2% 2,3,5-triphenyl tetrazolium chloride (TTC) for 20 min. Using image analysis software (ImageJ, version 1.53a), the non-infarcted area of the ipsilateral hemispheres and the areas of the contralateral hemisphere were calculated on the rostral side and caudal side, respectively.

$${\text{Infarct volume on each section }}\left( {{\text{mm}}^{{\text{3}}} } \right){\text{ }} = {\text{ }}({\text{areas of the contralateral hemisphere}} - {\text{the non-infarcted area of the ipsilateral hemisphere}}){\text{ ontherostral side }}\left( {{\text{mm}}^{{\text{2}}} } \right){\text{ }} \times {\text{ 1 }}\left( {{\text{mm}}} \right){\text{ }} + {\text{ }}({\text{areas of the contralateral hemisphere}} - {\text{the non-infarcted area of the ipsilateral hemisphere}}){\text{ onthecaudal side }}\left( {{\text{mm}}^{{\text{2}}} } \right){\text{ }} \times {\text{ 1 }}\left( {{\text{mm}}} \right).$$

The sum of each section volume is considered to be the total volume of infarction^29,30^. A postmortem analysis was performed by one researcher (H.M.), who was blinded from details of the surgical procedures.

### Ethics

Our study was approved by the Institutional Animal Care and Use Committee of the 26 Jikei University School of Medicine (protocol number: 2016-105).

## Results

**Figure 5:**
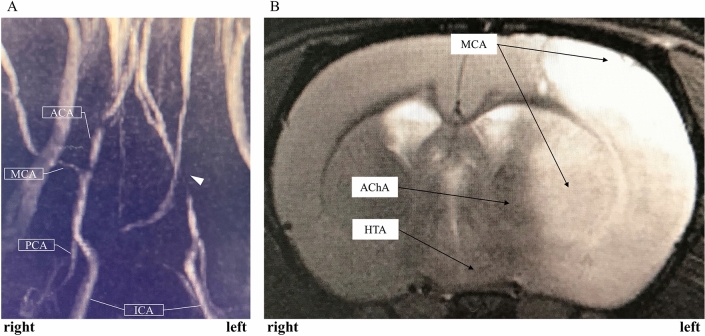
MRI 24 h after the creation of the zirconia MCA occlusion model. The zirconia ball did not affect the MRI. (**A**) MR angiography detected the impaired blood flow in the left MCA. (**B**) T2-weighted MRI showed infarction only in the MCA perfusion area, but not in the AchA and HTA. *AChA* anterior choroidal artery, *HTA* hypothalamic artery, *MCA* middle cerebral artery, *MRI* magnetic resonance imaging.

**Figure 6:**
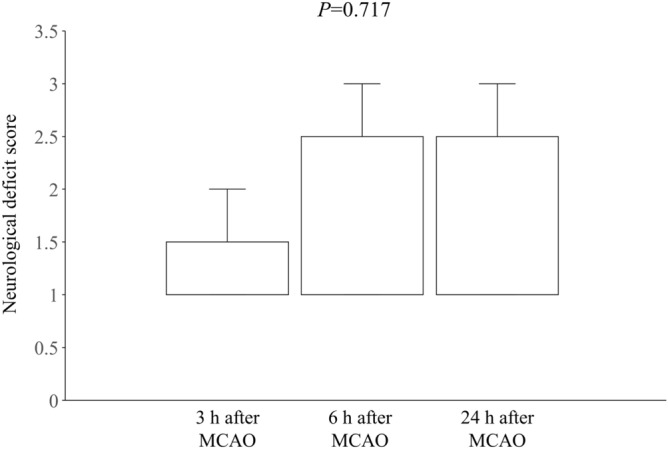
Box-plot shows neurological deficit score at 3, 6 and 24 h after MCAO. Neurological deficit scores were not statistically different at any time point (P = 0.717; Friedman’s test). *MCAO* occlusion of the middle cerebral artery.

**Figure 7:**
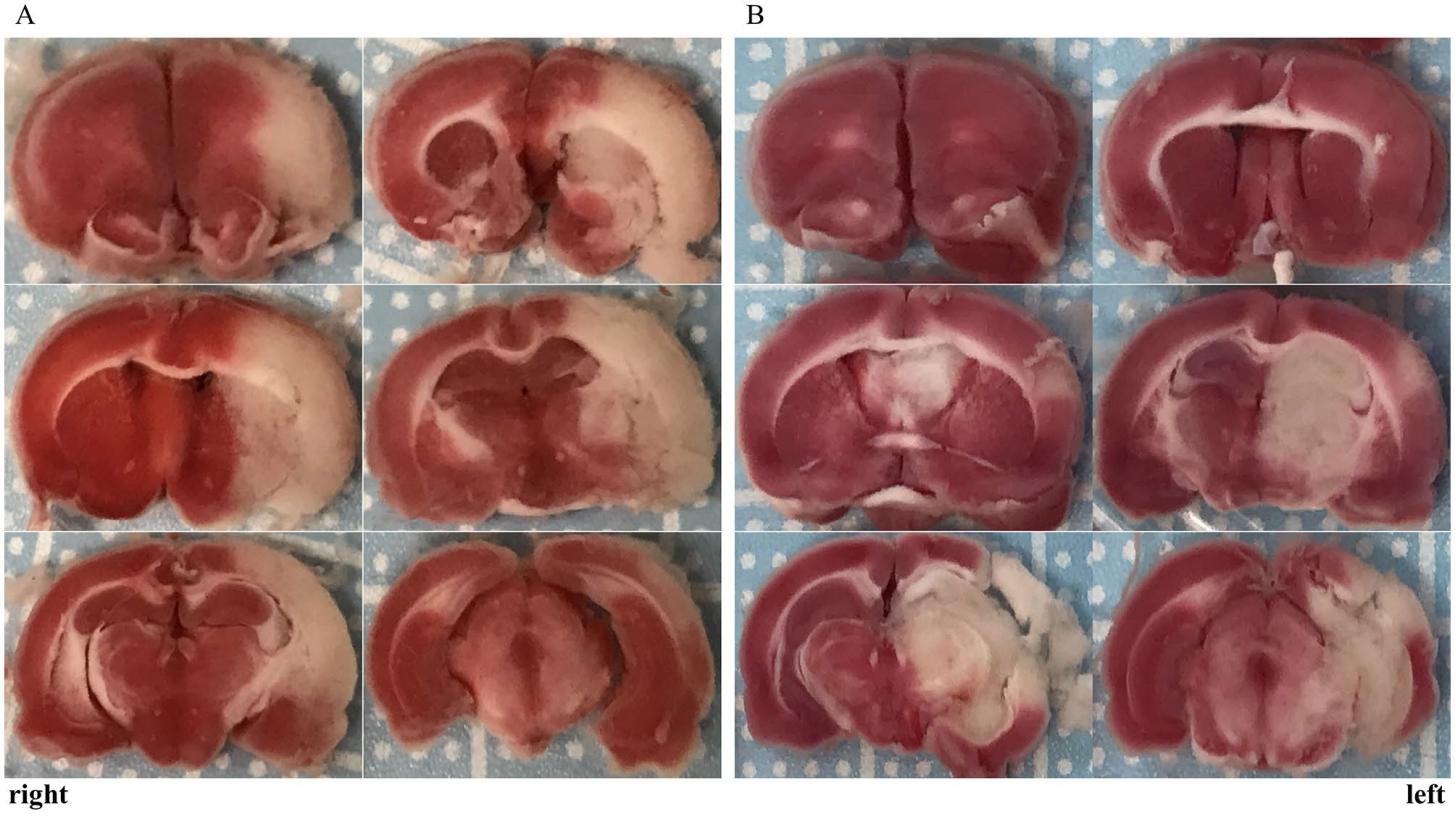
The brain is sliced and stained with 2% TTC solution 24 h after the left cerebral artery occlusion. (**A**) Brain infarction in the territory of only the MCA, but not in the AChA, VTA and HTA is clearly visible on TTC staining. (**B**) TTC staining showed that the left PCA territory changed post-infarction. Infarction of the hippocampus and substantia nigra were observed. *AChA* anterior choroidal artery, *HTA* hypothalamic artery, *MCA* middle cerebral artery, *PCA* posterior cerebral artery, *TTC* 2,3,5-triphenyltetrazolium chloride, *VTA* ventral thalamic artery.

All rat cerebral angiographies were successful. There no exclusions. No rats had hemorrhagic complications including a subarachnoid hemorrhage (SAH). Eight (80%) rats underwent occlusion of the ACA-MCA bifurcation by zirconia ball (Figs. 3B,C, 4B, Supplemental File—Video 1). Accidentally, the left PCA was failure embolized in 2 rats (20%) through the proximal PCA (Fig. 3D, Supplemental File—Video 2). Fluoroscopy was able to identify which the MCAO and model success while creating the model. The median operating time was 8 min (interquartile range [IQR] 6–14 min). The maximum contrast material dose was up to 1 ml, and the intraoperative blood loss was less than 1 ml. No cases of spontaneous recanalization 24 h after the embolization occurred. Cerebral angiography and MR angiography 24 h after the creation of the stroke model determine continuous MCAO. The zirconia ball did not affect the MRI (Fig. 5). No tails had ischemia because the 2 lateral caudal arteries took over the supply through numerous anastomoses. There were no deaths within the 24 h before the postmortem analysis. All MCAO rats revealed an incomplete hemiparesis on the right side with neurological deficit score ranging from 1 to 3 (median 1, IQR 1–3) at 24 h after the induction of ischemia. There was no statistical difference in neurological deficit score at 3, 6 and 24 h after MCAO (*P* = 0.717) (Fig. 6). Twenty-four hours after the left PCA occlusion, neurological deficit score was 3 and 4. Only one rat after PCA occlusion had seizures. Furthermore, 2% TTC staining showed that the median infarct volumes (mm^3^) were 280 (IQR 267–333) 24 h after the left MCA bifurcation occlusion (Fig. 7A). Twenty-four hours after the left PCA occlusion, the stroke lesion volume was 251 mm^3^ and 446 mm^3^ (Fig. 7B). There was no infarction of the thalamus and hypothalamus in MCAO model.

## Discussion

We have presented a novel zirconia MCAO rat model that is minimally invasive and highly reproducible. To the best of our knowledge, this is the first study to present a highly selective MCAO model by percutaneous caudal arterial puncture using zirconia ball and microcatheter under fluoroscopic guidance. Fluoroscopy was able to identify which the MCAO and model success while creating the model. It permitted exclusion of animals with complications from the experiment. The advantages of the zirconia MCAO rat model for experimental studies are discussed below and are summarized in Table 1.

First, this novel model can create an infarct only in the MCA territory without ischemia in the territories supplied by the ECA, the PCA, and branches of the ICA, and can also identify the site of vessel occlusion intraoperatively. All existing models of MCAO except caudal artery approach share the same disadvantages. Ligation of the ECA and insertion of the suture occludes the entire course of the ICA, leading to obstruction of the ECA, the PCA, and branches of the ICA, such as the hypothalamic and anterior choroidal arteries. Ligation of the ECA results in ischemic necrosis of the mastication and hypopharyngeal muscles, which negatively affects behavioral testing outcomes^12^. The PCA and branches of the ICA are the main arteries to the thalamus, hypothalamus, hippocampus, and substantia nigra^31–33^. As such, because these important areas are infarcted, the animal have a variable level of increased body temperature, disturbed water homeostasis, severe paresis, and high postoperative mortality, which altogether can result in bias in experimental studies^5–11^. The macrosphere and suture models lead to reduced blood flow in the ECA and anterior choroidal artery^14,15^. The zirconia MCAO model can reduce inter-animal variability and mortality by selectively causing ischemia only in the MCA blood flow area.

Second, the zirconia MCAO model is minimally invasive and safe as it only requires a caudal artery puncture and does not require a craniotomy and cervical incision (Table 1). Moreover, in our study, there was not a single case of hemorrhagic complications. SAH occurred in 8–30% of animals in the MCAO model by intraluminal suture method (Table 1)^16–18^. SAH can lead to serious complications such as vasospasm and intracerebral hemorrhage, and thus can confound the results of experimental stroke studies. If SAH occurs, the zirconia MCAO model can detect SAH while creating the model using fluoroscopy. Minimally invasive and safe methods can prevent the possible loss of the experimental animal, and lead to a “reduction” in the suffering and “refinement” of the welfare of laboratory animals^34^.

Third, the zirconia MCAO model uses fluoroscopy, which means that vessel occlusion and model success can be checked for in real-time while creating the model. This is particularly important for treatment intervention studies using animal models because it avoids performing an intervention with failed models.

Finally, by placing a plastic outer indwelling needle in the caudal artery, cerebral angiography can be repeated. This method can also be used for arterial drug administration, which can be repeated in a highly selective and direct manner to the target artery. Multiple doses of the drug or cells can be administered to the target organ at the time of therapeutic intervention, thereby enabling the establishment of more sophisticated and complex therapeutic intervention studies. Endovascular therapy is already commonly used in humans and is easy to apply clinically.

Our study has several limitations. First, the model failure rate was 20% due to the occlusion of PCA instead of MCA. Second, left ACA blood flow was dependent on the right ACA instead of the left ICA. However, no infarcts in the ACA region appeared, so this does not appear to be an issue. Third, although there was a lack of data on time course study of cerebral infarction, similar previous study has already shown that infarction gradually enlarge and become complete^14^. Fourthly, as for the availability of the radiological imaging equipment, it is unclear whether the average basic laboratory around the world owns the equipment or has the expertise in using it. Fifthly, it is clear that zirconia has biological safety and engineering stability^35–37^. However, we did not directly evaluate immunological responses to zirconium ball compared to conventional clots or biological emboli. Finally, the model cannot induce reperfusion. Any of the current preclinical model of stroke including the current one in this study has caveats. We think multidirectional approach using different preclinical models can help to better understand the pathophysiology of stroke.

## Conclusions

In conclusion, the zirconia MCAO model presented here has several advantages. It is minimally invasive, and fluoroscopic guidance allows for the selective endovascular occlusion of the MCA. The technique also avoids ischemia of the ECA, anterior choroidal artery, hypothalamic artery, and PCA. This is important because the confounding effects of tissue damage outside the MCA territory in the MCAO model are considerable. From an ethical standpoint, this model significantly limits the suffering caused by complications secondary to large, unspecific infarct lesions, surgical exploration of the neck, and ligation of the ECA. The microcatheter that carries the microwire can also serve as a working channel wherein therapeutic agents may be administered in a targeted fashion. We hope that this approach will be carried out by many researchers, leading to a “reduction” in the suffering and “refinement” of the welfare of laboratory animals. The development of the zirconia MCAO model also helps reduce costs associated with investing in unnecessary clinical trials, the death of laboratory animals, and ineffective drug discovery.

## Supplementary Information

Supplementary Legends.

Supplementary Video 1.

Supplementary Video 2.

## Abbreviations

ACA: Anterior cerebral artery
AchA: Anterior choroidal artery
CCA: Common carotid artery
ECA: External carotid artery
ICA: Internal carotid artery
IQR: Interquartile range
LAO: Left anterior oblique
MCA: Middle cerebral artery
MCAO: Occlusion of the middle cerebral artery
MRI: Magnetic resonance imaging
PCA: Posterior cerebral artery
TTC: 2,3,5-Triphenyl tetrazolium chloride
SAH: Subarachnoid hemorrhage
