# Supplementary material for: A novel model of ischemia in rats with middle cerebral artery occlusion using a microcatheter and zirconia ball under fluoroscopy
Source: Sci Rep. 2021 Jun 17;11:12806. doi: 10.1038/s41598-021-92321-w (PMC8211726; doi:10.1038/s41598-021-92321-w)
Supplement: Supplementary file 1 — Supplementary Legends. [file 41598_2021_92321_MOESM1_ESM.docx]

**Supplementary Video 1**

After the catheter was guided to the left internal carotid artery, one zirconia ball of 0.4 mm in diameter was advanced in the catheter by a slow injection of heparinized physiological saline, and the anterior cerebral artery–middle cerebral artery bifurcation was selectively embolized (left anterior oblique angle = 80°).

**Supplementary Video 2**

In the frontal view, after the catheter was guided to the left internal carotid artery, one zirconia ball of 0.4 mm in diameter was advanced in the catheter by a slow injection of heparinized physiological saline. The zirconia ball flowed into the middle cerebral artery origin, but then accidentally flowed back into the left posterior cerebral artery.
